# Multidirectional Shoulder Instability: Treatment

**DOI:** 10.2174/1874325001711010812

**Published:** 2017-08-31

**Authors:** Miguel Angel Ruiz Ibán, Jorge Díaz Heredia, Miguel García Navlet, Francisco Serrano, María Santos Oliete

**Affiliations:** 1 Hospital Universitario Ramon y Cajal, Cta Colmenar km9, 100, Madrid, 28046, Spain; 2 Hospital Asepeyo Coslada, Calle de Joaquín de Cárdenas, 2, 28823 Coslada, Madrid, Spain

**Keywords:** Shoulder, Multidirectional instability, Conservative treatment, Arthroscopic capsular plication, Open capsular shift

## Abstract

**Background:**

The treatment of multidirectional instability of the shoulder is complex. The surgeon should have a clear understanding of the role of hiperlaxity, anatomical variations, muscle misbalance and possible traumatic incidents in each patient.

**Methods:**

A review of the relevant literature was performed including indexed journals in English and Spanish. The review was focused in both surgical and conservative management of multidirectional shoulder instability.

**Results:**

Most patients with multidirectional instability will be best served with a period of conservative management with physical therapy; this should focus in restoring strength and balance of the dynamic stabilizers of the shoulder. The presence of a significant traumatic incident, anatomic alterations and psychological problems are widely considered to be poor prognostic factors for conservative treatment. Patients who do not show a favorable response after 3 months of conservative treatment seem to get no benefit from further physical therapy.

When conservative treatment fails, a surgical intervention is warranted. Both open capsular shift and arthroscopic capsular plication are considered to be the treatment of choice in these patients and have similar outcomes. Thermal or laser capsuloraphy is no longer recommended.

**Conclusion:**

Multidirectional instability is a complex problem. Conservative management with focus on strengthening and balancing of the dynamic shoulder stabilizers is the first alternative. Some patients will fare poorly and require either open or arthroscopic capsular plication.

## 1 INTRODUCTION

Multidirectional instability of the shoulder is a complex problem that is often difficult to diagnose and requires careful assessment prior to any treatment decisions are made. Many definitions have been proposed in the literature but the working definition proposed by Neer in 1980 [1], a shoulder that dislocates in at least two directions, is probably still valid today. The problem affects younger individuals, generally females and is relatively uncommon compared to traumatic instability [2].

The main issues regarding the relevant anatomy, the physical exam, imaging, physiopathology and natural history have been addressed elsewhere in this monographic issue and will not be reviewed here, but, having a clear understanding of the role of hiperlaxity, anatomical variations, muscle misbalance and possible traumatic incidents in a particular multidirectional instability patient, is key in planning an adequate treatment protocol.

Most patients with multidirectional instability will be best served with a period of conservative management with physical therapy that can be effective in the long term in more than half of the patients [3]. When conservative treatment fails, a surgical intervention is warranted. Many different surgical procedures have been proposed and outcomes have been wildly variable. Nowadays, both open capsular shift and arthroscopic capsular plication are considered to be the treatment of choice in these patients [4]. The purpose of this review is to present the different conservative and surgical treatment alternatives and analyze their outcomes.

## 2 CONSERVATIVE TREATMENT

Conservative treatment with physical therapy is going to be the initial treatment of choice for most patients with symptomatic multidirectional instability (MDI) of the shoulder [5-7]. Instability is due to a combination of defects of the static and dynamic stabilizers of the shoulder. A rehabilitation program will act upon the dynamic stabilizers with exercise therapy, but will also attempt to compensate for static deficiencies. The success of rehabilitation in a considerable amount of patients shows that improvement of both muscular capabilities through strengthening and neuromuscular control of the joint can achieve this compensation [8].

Bulkhead *et al.* showed good results in patients with MDI treated with a rehabilitation program based on strengthening exercises, especially on atraumatic cases. Poorer results were seen in shoulders with traumatic subluxation [5]. The results of nonsurgical treatment in a long term study done by Misamore *et al.* [3] in a young population of athletic patients was not so promising: after a mean follow-up of 8 years, one in three of the patients had required surgical treatment, one in three had persistent instability or pain and only half rated their shoulder function as better or much better after conservative treatment. Patients in these studies responded to the exercise program within 3 months. Most of the patients who did not show a favorable response in these first 3 months did not seem to improve in the following months in spite of persistent therapy [3, 5].

An electromyographic analysis performed after conservative treatment in patients with MDI showed how muscular control can be restored [9]. But results are not always good. The underlying pathology will determine the outcome after rehabilitation. In patients with post-traumatic instability, there may be ligament or bone lesions that will inevitably alter normal kinematics [10]. Some authors have found that psychological elements, work related injury and patients with previous shoulder surgery were less likely to obtain results from a rehabilitation program [6, 11].

Although there is a clear recommendation for exercise-based treatment in most patients, there is not enough evidence to support a specific exercise program. There is neither enough data on the types and rhythm of these exercises [12].

In the following section, the key points in the rehabilitation of MDI patients will be presented and a series of exercises that can be included in a rehabilitation program will be proposed. These are not limited to specific exercises described in MDI rehabilitation publications but exercises that have shown efficacy in the training of scapular stabilizers and rotator cuff muscles are also included.

The exercises should focus in four main areas: posture education, proprioception, flexibility and muscular training:

## 2.1 Education and Correction of Posture

Rehabilitation programs in MDI patients usually start by explaining the patient the problem concerning the shoulder and making them aware of their abnormal muscle patterns. Patients must be instructed on activity modification and the need to regain strength and neuromuscular coordination of scapular and rotator cuff muscles [6, 11, 13].

## 2.2 Proprioception

Proprioception is altered in patients with MDI of the shoulder. A study with MDI patients performing a series of upper limb tasks using a three-dimensional video motion analysis system showed significantly greater position errors in these patients compared to control subjects [8].

A rehabilitation program can start with posture-correction strategies and proprioceptive exercises to improve scapulothoracic and glenohumeral movement patterns [6, 14] Mirrors can be used for the correction of movement patterns [6]. In early stages, scapular retraction exercises can be done in a standing position to improve proprioception.

**Fig. (1):**
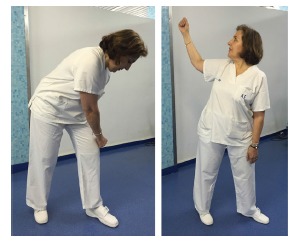
The Lawnmower. The exercise starts with legs flexed and trunk rotated towards the contralateral side extending the arm with the hand at the level of the patella. Then we rotate and extend legs and trunk to a vertical orientation while retracting the scapula with the elbow flexed.

The lawnmower exercise activates kinetic chains involving the trunk, hip and scapular stabilizers, training scapular control in open chain (Fig. **1**) [10].

**Fig. (2):**
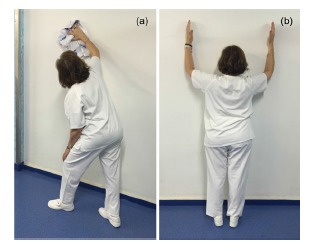
Wallslides. **(a)** Starting with legs and trunk flexed and holding a cloth, we push the cloth up the wall as we extend legs and trunk. **(b)** Stand facing a wall placing the ulnar border of both hands touching the wall. Gently push into the wall and slide your hands upwards.

Closed chain exercises like the wall slides activate the serratus anterior in positions above 90° of humeral elevation and can be performed adding kinetic chain component with trunk and hip flexion/extension (Fig. **2**) [10, 15].

Occupational therapy can be useful to promote the functional capacity of the shoulder with task related activities [6].

## 2.3 Flexibility Exercises

**Fig. (3):**
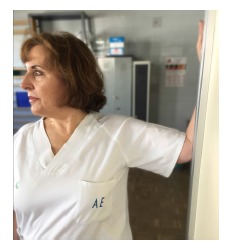
Corner stretch: Place the hand on the corner of a pillar with the shoulder abducted 90º and the elbow flexed 90º. Then we rotate the trunk away from the shoulder until stretching of the pectoral muscles.

Pectoralis minor and short head of the biceps contracture can contribute to scapular dyskinesis by producing scapular protraction. Also, latissimus dorsi activation can be a key destabilizing force, pulling the humeral head inferiorly [10]. Flexibility exercises should focus on preventing tightness of these muscles. The corner stretch self-stretch was found by Borstad *et al.* as a suitable exercise for elongation of the pectoralis minor muscle (Fig. **3**) [16].

### 2.4 Muscle Training

Patients with MDI have abnormal shoulder kinematics when compared with healthy control subjects [17, 18]. There is electromyographic evidence of abnormal patterns of muscle activation in these patients [19]; the abnormalities are complex and usually affect the coordination and strength of both the rotator cuff muscles and the scapulothoracic stabilizers [6].

## 2.5 Scapular Stabilizers

Muscles surrounding the scapula should be carefully assessed during physical therapy as these are key in placing the glenohumeral joint in space and avoiding instability, as subtle alterations in glenoid plane orientation are an important component of glenohumeral stability [7]. Scapular dyskinesis altering the normal shoulder biomechanics is frequently observed in patients with glenohumeral instability [10]. Dyskinesis in a patient with shoulder instability is the result of inhibition of coordinated muscle activation, muscle fatigue, muscle inflexibility and learned compensation patterns [20]. Dyskinesis produces increased anterior tilt, internal rotation and scapular protraction, decreasing rotator cuff activation [10]. Also a decreased upward rotation is produced contributing to inferior glenohumeral joint instability [21].

Strengthening programs should start with the scapular stabilizers, including the lower trapezius, rhomboids, and serratus anterior.

**Fig. (4):**
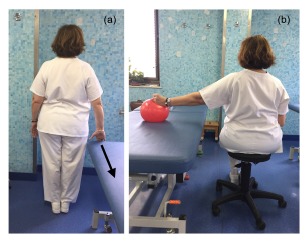
**(a)** Low row. Standing up, we place the hand on the anterior edge of a solid surface and push against it as we retract and depress the scapula. **(b)** Inferior glide. Sitting beside a surface with the arm abducted and elbow extended, we apply pressure adducting the shoulder and depressing the scapula.

The low row and the inferior glide are isometric exercises that activate the lower trapezius and serratus anterior, two key muscles for scapular stability [10]. Rowing exercises can be added for more intense training (Fig. **4**) [22].

**Fig. (5):**
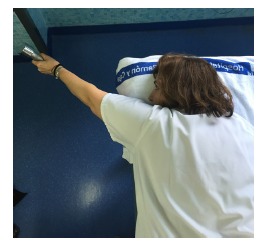
Blackburn “Y” exercise. In prone position we perform a horizontal abduction at 125º with arm in full external rotation.

The Blackburn exercises are optimal for stimulation of lower trapezius being the “Y” the optimal exercise for this purpose (Fig. **5**). This exercise is performed abducting the arm overhead in 120º in external rotation with the patient in prone position [23].

**Fig. (6):**
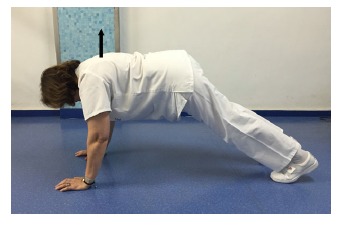
Push up plus. Start the exercise in the push up position with hands shoulder-width apart. With the elbows extended perform a maximal scapular protraction.

The push up plus exercise activates the serratus anterior muscle with minimal activation of upper trapezius (Fig. **6**). The serratus anterior muscle is key as it is the only scapulothoracic muscle that produces upward rotation of the scapular with AC joint external rotation and posterior tilting [21, 24].

Strengthening of the posterior part of deltoid and triceps muscles is also recommended because they play an important role in the stabilization of the glenohumeral joint [25].

## 2.6 Rotator Cuff Muscles

Different studies have shown that patients with MDI have asynchronous patterns of rotator cuff muscle activation [26]. This supports the association of MDI with neuromuscular control deficiency affecting the rotator cuff. Training exercises should be prescribed to help center the humeral head within the glenoid fossa during shoulder elevation [18, 19]. Rotator cuff strengthening exercises can be added to the rehabilitation program once proper scapulothoracic control is achieved.

**Fig. (7):**
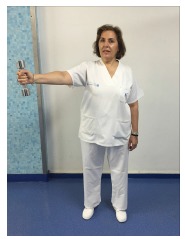
Full can. Standing up and holding a dumbbell, we perform an abduction with the shoulder in external rotation and elbow extended. With the arm slightly in front of the trunk, raise the arm to shoulder height.

**Fig. (8):**
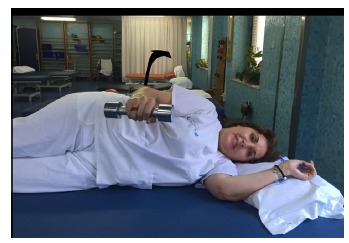
External rotation side lying. Laying on our side holding a dumbbell, we perform an external rotation with the elbow flexed.

Apart from Blackburn exercises, classic rotator cuff exercises can be performed to strengthen these muscles. The horizontal abduction with external rotation exercise (“full can” position) is the best way to strengthen the supraspinatus muscle (Fig. **7**) [24, 27]. The exercise that elicits the most combined EMG signal for the infraspinatus and teres minor is the side-lying external rotation (Fig. **8**) [22, 27].

## 3 SURGICAL TREATMENT

The fact that multidirectional instability is a problem that affects young adults and adolescent but is seldom found in older adults suggest that some patients might heal without an specific treatment and at least more than half will benefit greatly from conservative treatment [3], some patients will still require surgery to alleviate their instability, pain or loss of function. The pioneering work by Neer [1] inspired a host of surgeons in performing open capsular shifts in these patients; a procedure that has been found to be highly successful [28]. The advent of arthroscopy in the late eighties and nineties allowed for a new understanding of the capsular anatomy of the shoulder “from inside”, and prompted a new breed of skilled arhtroscopists to try to reproduce the open procedures through the “keyhole” [29]. Less than a decade later, these same surgeons found that a new available tool, thermal capsuloraphy might be the key of a more balanced restoration of function in the multidrectionally unstable shoulder [30]. Although arthroscopic capsular plication is extremely popular and is on its way to be considered the new gold standard, thermal capsuloraphy faded into oblivion due to unacceptable tissue loss and skyhigh recurrence rates [31].

The question of whether open or arthroscopic procedures are the best option in the multidirectionally unstable patient is yet unsolved. Longo *et al.* in 2015 performed a systematic review focused in defining the best available treatment option in these patients [4]. Arthroscopic capsular plication and open capsular shift were the best surgical procedures for treatment of MDI after failure of rehabilitative management and that arthroscopic capsular plication showed results comparable to open capsular shift. 21% of patients undergoing physiotherapy required surgical intervention, whereas the overall occurrence of redislocation was seen in 10% of shoulders undergoing surgical procedures. The redislocation rate was 7.5% for open capsular shift, 7.8% for arthroscopic plication, 24.5% for arthroscopic thermal shrinkage, and 22% for arthroscopic laser assisted capsulorrhaphy.

Chen *et al.* performed a meta-analysis in 2016 that compared the open approach, arthroscopic and thermal capsular shrinkage [32]. They found rates of recurrence of 9.9% (7.3-12.9%) for the open technique and 6.08% (3.7-8.9%) for the arthroscopic technique. Thermal shrinkage was found to have a high rate of recurrence of 23.9% (16.6-32.2%). Both open and arthroscopic approaches showed low reoperation rates and few complications. Loss of range of motion was found to be the main difference. Open procedures had loss of motion rates of 33% (27.7–40.1%), whereas arthroscopic procedures caused stiffness in only 5.5% (3.6–9.8%) of cases. External rotation loss is the most pronounced sign among these two groups. After open procedures, the mean loss of external rotation with arm at side is 7.0 (3.3–10.6) degrees, and 2.0 (0.9–2.4) degrees in the arthroscopic group.

### 3.1 Open Procedures: Inferior Capsular Shift

The open approach of multidirectional instability was the standard surgical technique used until the expansion of the arthroscopy during the end of the 1990s [33]. Since the development of arthroscopic techniques during the beginning of the 21st century, its use has drastically declined [4]. However, it is a technique that has shown good results in the long follow up and it is considered the gold standard against arthroscopic techniques are evaluated [4, 32, 33].

The goal of surgery is to perform a plication of the inferior capsule that results in a reduction of the anterior, inferior and posterior capsular volume. Plication of the inferior capsule is designed to allow the stretching of the capsule on the more involve approach side, and tensioning the capsule of the inferior and opposite side.

After a thorough examination under anaesthesia to elucidate the dominant direction of instability, the patient is placed in a beach-chair position for a planned anterior approach or lateral decubitus for a posterior approach. Shoulders that dislocate both anteriorly and posteriorly should be approached from the anterior side. Cooper *et al.* [34] have preferred to approach all cases from the anterior aspect of the shoulder. The reasoning behind this is that the rotator-cuff interval cannot be closed from the back, and reasonable posterior tightening can be obtained from the front.

A classical deltopectoral approach or an axillary approach [28], that has the advantage of being more cosmetic, is made; the interval between the deltoid and pectoralis major is identified and the cephalic vein is protected. The conjoined tendon is retracted medially and the insertion of the subscapular tendon is identified. Careful management of the subscapularis tendon is a fundamental step of the surgical technique for a proper functional outcome. The subscapularis tendon can be managed in three different ways: a.- performing a complete vertical tenotomy 0.5 to 1 cm medial to the lesser tuberosity; b.- performing an inverted L-shaped tenotomy; or c.- longitudinally splitting the muscle at the junction of the middle and inferior thirds of the subscapular tendon. Anyway, either the subscapularis tenotomy or the subscapularis split should allow for adequate visualization of the anterior capsule.

The capsular incision to access the joint may be performed laterally (humeral) as described by Neer and Foster [1] intermediate (middle) as advocated by Wirth [35] or medially (glenoid) as described by Altchek [36]. The lateral approach has some advantages over other approaches [28]: it exposes a greater amount of capsular tissue, allows for a greater degree of plication and the risk of axillary nerve injury is lower.

**Fig. (9):**
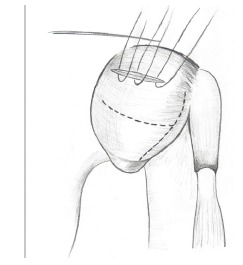
The interval between the superior and middle glenohumeral ligaments should be closed with nonabsorbable sutures.

**Fig. (10):**
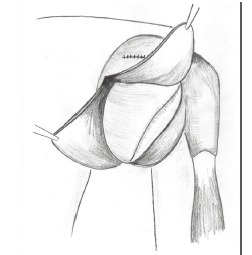
The capsule is incised using a humeral-based T shape incision between the middle and inferior glenohumeral ligaments.

**Fig. (11):**
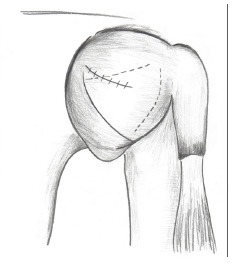
The inferior flap is mobilized and pulls up superiorly, and fixed to the remnant lateral capsular tag or adjacent subscapularis stump. The superior flap is pulled down laterally and inferiorly and sutured on the inferior flap.

The interval between the superior and middle glenohumeral ligaments should be closed with nonabsorbable sutures (Fig. **9**) [37]. The capsule is incised using a humeral-based T shape incision between the middle and inferior glenohumeral ligaments, with the upper bar of the “T” placing lateral and oriented longitudinally (Fig. **10**) [36]. Stay sutures are placed in both flaps of the capsule as they are mobilized. As the humerus is externally rotated and flexed, the capsule is incised around the neck of the humerus, extending as far posteriorly as necessary depending on the degree of instability [28]. The inferior flap is mobilized and pulls up superiorly until the inferior recess is obliterated and fixed to the remnant lateral capsular tag or adjacent subscapularis stump with 2.0 non-absorbable sutures while the arm is positioned in 45 degrees of abduction and 10 º external rotation. After that, the arm position is changed to adduction as the superior flap is pulled down laterally and inferiorly and sutured on the inferior flap (Fig. **11**) [1]. The subscapularis muscle is reattached to its insertion in an anatomic manner. The subcutaneous tissue and the skin are closed in layers. The arm is immobilized in a sling on slight abduction and external rotation during 6 weeks. Passive range of motion is started in the first postoperative days, except rotations that are restricted during 4 weeks.

If the dominant direction of instability is posterior, the capsular shift is made with a posterior approach. The deltoid muscle is detached from the acromion and scapular spine. The infraspinatus is peeled medially and the posterior capsule is exposed. The plication of the capsule is made in the same way of the anterior approach.

Cordasco in 2000 reviewed the available literature and found that this open technique has high rates of satisfaction with low rate of recurrence of instability [28].

### 3.2 Artrhoscopic Procedures: Capsular Plication

Any surgical treatment in the setting of a shoulder instability should begin with a thorough examination under anesthesia, in order to address the real amount of shoulder laxity in every direction. More so in those patients in which clinical examination was compromised by excessive pain and guarding. In multidirectional instability (MDI) this becomes of paramount importance. It should be done in comparison with the contralateral shoulder and correlating the findings with the preoperative information from the patient´s history, imaging studies and physical exam. By doing so we will be able to have a better understanding of the real amount of displacement and its direction in order to plan the surgical procedure.

**Fig. (12):**
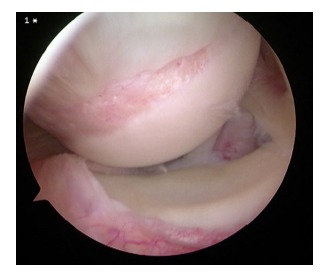
Arthroscopic view of a typical multidirectional instability in a left shoulder obtained from the posterior viewing portal . The laxity is patent and a “full view” of the glenoid surface is possible.

**Fig. (13):**
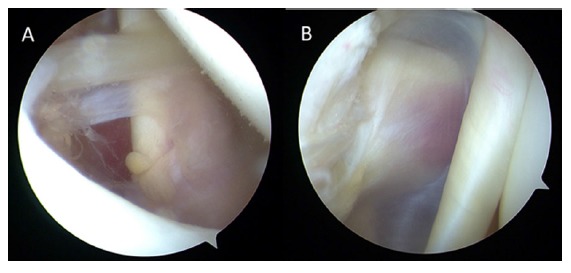
Arthroscopic view of the anterior aspect of a right shoulder with multidirectional instability. There is clear labral absence **(A)**, medial glenohumeral ligament atrophy **(A)** and anterior capsular atrophy that allows a “see through” view of the coracobraquialis tendons from inside the joint **(B)**.

The arthroscopic procedure has become the preferred method for surgical treatment, since it allows for a less aggressive approach to the static stabilizers without disturbing any of the dynamic stabilizers, (*e.g.*, subscapularis tendon *via* a partial tenotomy or split during an anterior open approach). Since the most widely accepted pathological findings in MDI are a redundant and patulous postero-inferior and inferior capsule [38], arthroscopy provides another great advantage by allowing a better visualization and confirmation of the associated capsulolabral alterations that would reduce the effective glenoid depth, like posteroinferior labral retroversion (Figs. **12** and **13**) [39].

Arthroscopy can be performed either in lateral decubitus with a 30º of abduction, slight forward flexion of 10º to 15º and a traction of 5 kg applied, which is our preferred method, or in a beach chair position. It is recommended that the patient should be prepped to allow for traction release and elbow flexion during the surgical procedure.

The procedure is started through a posterior portal, which we place more lateral and distal than the standard posterior viewing portal, in order to gain a better access to the postero-inferior structures. An initial diagnostic arthroscopy is performed, typically showing a significant drive through sign and capsular redundancy.

A systematic evaluation of all the capsulolabral structures is essential to define the main altered structures to be repaired; in order to do so an anterior and anterosuperior portal are performed, allowing us to place the camera in all three portals, thus obtaining a complete visualization of all relevant structures.

**Fig. (14):**
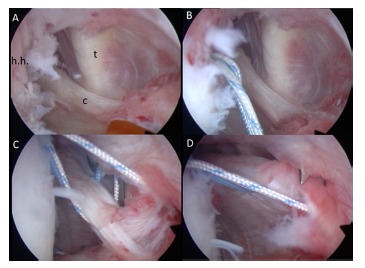
Repair of a reverse humeral avulsion of the glenohumeral ligamants: view from the anterior-superior portal of a right shoulder. **(A)** there is a capsular deficiency that allows for a clear view of the teres minor tendon (t). the capsule (c) is detached form its natural insertion in the humeral head (h.h.). **(B)** an implant is placed in the original insertion of the capsule in the humeral head. **(C)** sutures are passed through the capsule. **(D)** the capsule is restored to its original insertion.

It is important to highlight the need to address the capsular insertions not only at the glenoid side but at the humeral side as well; this is important to avoid missing a humeral avulsion of the glenohumeral ligaments (HAGHL) at the inferior pouch or at the posterior side (reverse HAGHL or a RHAGHL, (Fig. **14**)), and to rule out a Hill-Sachs lesion, that sometimes may be small but associated with a significant disruption of the capsular attachment.

Once the complete assessment is done and we have an idea of what structures are to be repaired, we typically use the anterosuperior portal as a viewing portal and the anterior and posterior ones as working portals.

**Fig. (15):**
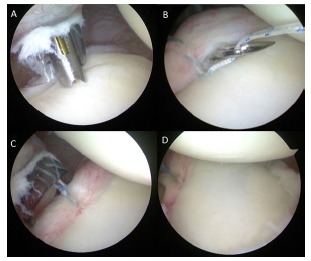
Arthroscopic view of the posterior part of a left shoulder with multidirectional instability obtained from the anterosuperior viewing portal. The objective is associating a posteroinferior plication **(A** to **C)** with restoration of labral height and centering of the humeral head after an associated anteroinferior plication **(D)**.

Starting at the posterior most side of the lesion the capsulolabral insertion is abraded with a rasp all the way to a healthy area, if any, in the anterior or anterosuperior side. In the absence of labral tears it´s been said that labral attachment is sufficient to plicate the capsule, as the healthy labrum has a biomechanical load to failure similar to a suture anchor [40], but concern about the real status of the labrum in the MDI patient regardless a good appearance together with the need to augment the labral height in many cases, because of the labral retroversion [41], makes us prefer the use of suture anchors, ideally the newest “all-suture” anchors with very small diameters, that allows for suture implantation with minimal aggression to the glenoid or the undetached labrum (Fig. **15**).

The capsular reconstruction sequence should be related to the primary direction of the instability, but considering that most will need an inferior capsuloplasty [38], and the fact that reducing the capsular volume will decrease the working and visualization area, plication will typically start at the inferior part, placing the postero-inferior anchor first. Grasping the capsule and make tensioning trials will be of great help in deciding where to place the anchors and how much capsule should be plicated.

Plication of the capsule in a nip and tuck fashion is sometimes needed, in order to reduce the capsular volume. We should aim to reproduce the anterior and posterior bands of the inferior glenohumeral ligament while augmenting any height deficit of the labral structure, at the postero-inferior side of the glenoid, but also important at the antero-inferior side, where a good bumper and not only a labral repair should be obtained.

By alternating suture anchor placement from posterior to anterior and so on, the complete capsular plication and labral reconstruction is performed as planned, paying special attention to the glenohumeral ligaments´ attachment, specially the medial attachments, and rule out any anatomical variation in this area that may increase the ligament's instability more so when associated to long head of the biceps insertion pathology (*e.g.*, Bufford complex plus SLAP lesion).

If, during arthroscopic examination, we find any humeral detachment of the posterior capsule or a Hill Sachs lesion, we will address this in first place, placing the anchors and passing the sutures as needed but leaving the knot tying for the end, since the closure of the posterior capsule will hamper the procedure.

The debate about the rotator interval´s role in MDI is still on [38, 42, 43]. and so is the need for an interval closure during MDI surgical treatment, but biomechanical [44] and some clinical studies [45] have shown the benefits of the interval´s closure. As we understand the anatomical basis for the interval´s closure, the superior glenohumeral ligament (SGHL) should be responsible for that potential benefit, so the closure of the interval should be performed in a manner that allows to repair this structure to its insertion site. Then, according to Farber´s biomechanical study [46], we perform this closure from medial to lateral in line with the fibers of the SGHL.

## CONCLUSION

Treatment of the multidirectionally unstable shoulder should be based in a clear understanding of the role of hiperlaxity, anatomical variations, muscle imbalance and possible traumatic incidents in each patient.

Conservative treatment is the best initial alternative in most patients and should be focused in improving posture, proprioception, flexibility and muscle strength and balance.

If rehabilitative management fails, arthroscopic capsular plication and open capsular shift are the best surgical procedures for treatment of these patients and have shown similar results.
